# An autophagosome-based therapeutic vaccine for HBV infection: a preclinical evaluation

**DOI:** 10.1186/s12967-014-0361-4

**Published:** 2014-12-20

**Authors:** Meng Xue, Fei Fan, Lei Ding, Jingyu Liu, Shu Su, Pengfei Yin, Meng Cao, Wei Zhao, Hong-ming Hu, Lixin Wang

**Affiliations:** Department of Microbiology and Immunology, Medical School of Southeast University, Nanjing, Jiangsu PR China; Cancer Research and Biotherapy Center, the Second Affiliated Hospital of Southeast University, Nanjing, Jiangsu PR China; Laboratory of Cancer Immunobiology, Earle A. Chiles Research Institute, Providence Portland Medical Center, Portland, Oregon USA

**Keywords:** Hepatitis B virus, DRibbles, Therapeutic vaccine, Autophagy

## Abstract

**Background:**

For more than 240 million chronic HBV carriers worldwide, effective therapeutic HBV vaccines are urgently needed. Recently, we demonstrated that autophagosomes were efficient antigens carriers and capable to cross-prime robust T-cell responses and mediate regression of multiple established tumors. Here we tested whether autophagosomes derived from HBV expressing cells could also function as a therapeutic vaccine.

**Methods:**

We generated an autophagosome-based HBV vaccine from HBV-expressing hepatoma cells and examined its ability to induce polyvalent anti-HBV T-cell responses and therapeutic efficacy in mouse models that mimic acute and chronic HBV infection in human.

**Results:**

When compared to the vaccine based on recombinant HBsAg, autophagosome-based HBV vaccine cross-primed multi-specific anti-HBV T-cell responses and significantly reduced HBV replication and HBcAg expression in livers of both acute and chronic mouse models. Therapeutic effect of this HBV vaccine depended on anti-HBV CD8^+^ effector T cells and associated with increased HBsAg and HBcAg specific IFN-γ producing T cells in the chronic mouse model.

**Conclusions:**

These results indicated that autophagosome-based HBV vaccine could effectively suppress the HBV replication, clear the HBV infected hepatocytes, and break the HBV tolerance in mouse model. The potential clinical application of autophagosome-based HBV vaccine is discussed.

**Electronic supplementary material:**

The online version of this article (doi:10.1186/s12967-014-0361-4) contains supplementary material, which is available to authorized users.

## Background

Chronic hepatitis B virus (HBV) infection remains a major health problem worldwide as it often causes many serious complications like hepatic cirrhosis and hepatocellular carcinoma [1]. The current treatment for chronic HBV infection is primarily based on antiviral chemotherapy or interferon therapy. Although these agents have improved considerably over the last 10 years, they fail to eradicate infection due to the persistence of HBV covalently closed circular DNA (cccDNA) in hepatocytes and the emergence of resistant viruses [2-5].

Therapeutic vaccination is a promising strategy to control chronic infection and delay the progression of diseases, especially when used in combination with antivial chemotherapy. Unfortunately, therapeutic vaccination of chronic HBV patients with HBsAg in combination with antiviral chemotherapy did not show any superior efficacy over chemotherapy alone [6]. Attempt to increase the therapeutic efficacy using HBsAg combined with anti-HBs immunoglobulin only showed a minor improvement [7]. Similarly, a vaccination strategy using DNA prime and protein boost also worked poorly in a recent randomized clinical trial [8]. These rather disappointing results are likely related to the fact that vaccination with the HBsAg alone is insufficient and a strong, polyvalent, and poly-functional CD8^+^ T-cell response against other HBV antigen besides HBsAg is required for viral clearance. Such a T-cell response was detected in subjects with self-limited HBV infection, but it is exhausted and functionally impaired in patients with chronic HBV infection [9]. Thus, novel therapeutic vaccines are urgently needed for the treatment of chronic HBV infection [10]. Based on our current knowledge, an ideal HBV therapeutic vaccine needs to be able to elicit a strong and multi-specific T cell response, which is capable of controlling HBV infection [11].

Recently, others and we have shown that autophagy of tumor cells or virus-infected cells plays important roles for the efficient cross-presentation of tumor and viral antigens [12-16]. However, cross-presentation favors abundant proteins with a long half-life and largely ignores short-lived proteins. The efficiency of cross-presentation of short-lived proteins could be greatly increased by inducing autophagy via inhibition of proteasome-mediated proteolysis. Consequently, autophagosomes containing short-lived defective ribosomal products (DRiPs), which we referred to as DRibbles, were found to be highly effective therapeutic cancer vaccines in multiple mouse cancer models [13-16]. Thus, it is generally accepted that autophagy can promote both MHC class II and class I restricted T-cell immune responses to either tumors or infectious pathogens [12-16].

Based on above findings, we hypothesized that increased autophagy of HBV expressing hepatoma cells [17] could enhance the sequestration of multiple HBV antigens into autophagosomes. These isolated autophagosome (HBV^+^ DRibbles) can serve as a potent vaccine to stimulate polyvalent HBV-specific T-cell responses. To test this hypothesis, we examined whether different autophagy inducers could increase the production of DRibbles from a HBV-producing hepatoma cells and further investigated whether HBV^+^ DRibbles vaccine could induce polyvalent anti-HBV immune responses and reduce ‘HBV infection’ in a mouse model [18].

## Materials and methods

### Ethics statement

All experimental protocols were approved by the Institutional Animal Care and Use Committee of Southeast University.

### Mice, cell lines and reagents

C57BL/6 female mice were purchased from the Comparative Medicine Center, Yangzhou University. All mice were bred and maintained in specific pathogen-free conditions. HepG2.2.15 and HepG2 cell lines were gifts from Dr. Jianqiong Zhang (Medical School of Southeast University, China). All the cells were cultured in complete medium made of DMEM or RPMI 1640 (Gibco, USA) supplemented with 10% heat-inactivated FCS (Hyclone, USA), 100 U/ml penicillin, 0.1 mg/ml streptomycin (Beyotime Institute of Biotechnology, China).

### HBV infection model

Naïve C57BL/6 mice (female, 6–8 weeks old) were injected with 10 μg of pAAV/HBV1.2 plasmid DNA (containing the HBV full-length genomic DNA) via the hydrodynamic injection as described [18].

### Preparation of HBV^+^ DRibbles or HBV^−^ DRibbles

HepG2.2.15 cells containing transfected HBV full-length DNA [17] or HepG2 cells were treated with 200 nmol/L Bortizomib (Millennium pharmaceuticals, USA) alone or in combination with 100 nmol/L Rapamycin (Enzo Life Sciences, China) or 30 mmol/L NH_4_Cl for 18 h, DRibbles containing autophagosomes were prepared from the culture media as described [13,14]. Morphology analysis of HBV^+^ DRibbles was done under transmission electron microscopy. HBsAg in HepG2.2.15 cell lysates was detected by western blot analysis using anti-HBsAg antibody (Santa Cruz, Biotechnology, Inc., USA). LC3 in both HepG2.2.15 cell lysates and HBV^+^ DRibbles was determined by western blot analysis using the polyclonal LC3B antibody (Cell Signal Technology, USA,1:1000), the HRP-labeled goat anti-rabbit IgG secondary antibody (1:5000) and a chemiluminescence kit (Multisciences Biotech Co., Ltd., China). Levels of HBsAg and HBeAg in HBV^+^ DRibbles were measured by ELISA (Shanghai Kehua Bioengineering Co., Ltd., China).

### Measurement of immune responses induced by vaccination with HBV^+^ DRibbles

C57BL/6 mice were immunized with different doses of HBV^+^ DRibbles or HBV^−^ DRibbles (100, 30, 10 μg total protein per mouse) or PBS via intranodal injection. Seven days later, 2 × 10^5^ lymphocytes per well were harvested and re-stimulated with HBV antigens or peptides (HBc129-140: PPAYRPPNAPIL; HBs190-197: VWLSVIWM) (ChinaPeptides Co., Ltd., China) for 24 h. The number of IFN-γ producing cells was detected by ELISPOT assay (Laizee Biotech Co., Ltd., China).

To determine whether HBV^+^ DRibbles elicited cell response and killed HBV infected hepatocytes, lymphocytes were harvested from HBV^+^ DRibbles vaccinated mice at day 7 and used as effector T cells; hepatocytes were collected from mice injected with pAAV/HBV1.2 plasmid DNA as the HBV-expressing target cells. Lymphocytes (2 × 10^6^/well) were co-incubated with target cells (2 × 10^4^/well). The supernatants were collected for detection of AST by clinical chemistry analyzer after 24 h or for detection of IFN-γ by ELISA (eBioscience, USA) after 72 h.

### Anti-HBV effect of vaccination with HBV^+^ DRibbles

C57BL/6 mice were immunized with HBV^+^ DRibbles, HBV^−^ DRibbles or PBS via intranodal injection as described above. Seven days later, 10 μg of pAAV/HBV1.2 plasmid DNA was injected via tail vein [18]. Serum samples were collected at day 14 for detection of HBeAg by ELISA and HBV genomic DNA with real-time PCR. Liver tissues were collected and embedded in paraffin. Intracellular HBcAg expression was detected by immunohistochemical staining with the rabbit anti-HBcAg antibody (1:100) using two-step method (Zhongshan Goldenbridge Biotechnology Co., Ltd., China). Percentages of HBcAg^+^ hepatocytes were calculated by counting at least 500 cells in five vision fields by two investigators for each sample.

### Depletion of CD4^+^ and CD8^+^ T cell subsets *in vivo*

Mice were vaccinated with 30 μg HBV^+^ DRibbles or PBS as above mentioned. On day 7, after injection of pAAV/HBV1.2 DNA into the mice, 200 μg anti-CD4 mAb (clone GK1.5, rat IgG2b) or/and 20 μg anti-CD8 mAb (clone 2.43, rat IgG2b) were administered intraperitoneally, and the depleted condition was maintained by repeated injections of the monoclonal antibody every 3 days for total of 3 injections. At day 14, HBeAg level and HBV DNA copy number in the serum samples and intrahepatic HBcAg expression in liver tissues were detected as described above.

### Therapeutic vaccination experiments

C57BL/6 mice were injected hydrodynamically with pAAV/HBV1.2 plasmid DNA and were vaccinated with 30 μg HBV^+^ DRibbles, 2 μg HBsAg with Al(OH)_3_ adjuvant (Center for Disease Control of Jiangsu Province, China) or PBS via intranodal injection 2 days later. Then, two intramuscular boost injections (2 μg of HBsAg with Al(OH)_3_ adjuvant or 30 μg of HBV^+^ DRibbles with αAl_2_O_3_ nanoparticles adjuvant [19]) were performed on day 5 and 7. The lymphocytes were isolated 7 days after first vaccination and re-stimulated with recombinant HBV antigens and peptides as described. The number of IFN-γ producing cells in 2 × 10^5^ lymphocytes was detected by ELISPOT. The serum samples were collected on day 7 and 14 after first vaccination for measurement of ALT, AST, HBeAg , HBsAg, HBV DNA. The serum anti-HBsAg antibodies were detected by ELISA on day 14 after first vaccination. Liver tissues were collected on day 14 after first immunization for detection of percentage of HBcAg^+^ hepatocytes as mentioned above. The liver sections were stained with hematoxylin-eosin.

### Adoptive transfer experiments

C57BL/6 mice were injected hydrodynamically with 10 μg pAAV/HBV1.2 plasmid DNA to establish HBV tolerant mice [18]. Sixty days later, tolerance toward HBsAg was noted in HBV carrier mice. The HBsAg tolerant mice were selected and then immunized with above HBV^+^ DRibbles or HBsAg or PBS on day 61, respectively. Two intramuscular boost injections with the same dose of vaccines as above were done on day 63 and 65. Lymphocytes were harvested from the immunized mice and re-stimulated with HBV antigens and peptides on day 67 as described. The number of IFN-γ producing cells in 2 × 10^5^ lymphocytes was measured by ELISPOT. In addition, at day 68, a total of 1 × 10^8^ lymphocytes from vaccinated HBsAg tolerant mice were adoptive transferred intravenously into each of the new set of 60-day HBV carrier mice. Serum samples were collected on day 3, 7, 14 and 21 after adoptive transfer for detection of ALT, AST, HBsAg and HBV DNA. The serum anti-HBsAg antibodies were measured by ELISA on day 21 after adoptive transfer. Liver tissues were collected on day 21 for detection of percentage of HBcAg^+^ hepatocytes. The liver sections were stained with hematoxylin-eosin.

### Statistical analysis

One-way ANOVA or two-way ANOVN with Bonferronic post test and the two-tails paired *t* test were performed using GraphPad Prism5.0, (GraphPad Software Inc., San Diego, CA). A *P* value of <0.05 was considered statistically significant.

## Results

### DRibbles are capable to encapsulate multiple HBV antigens

**Figure 1:**
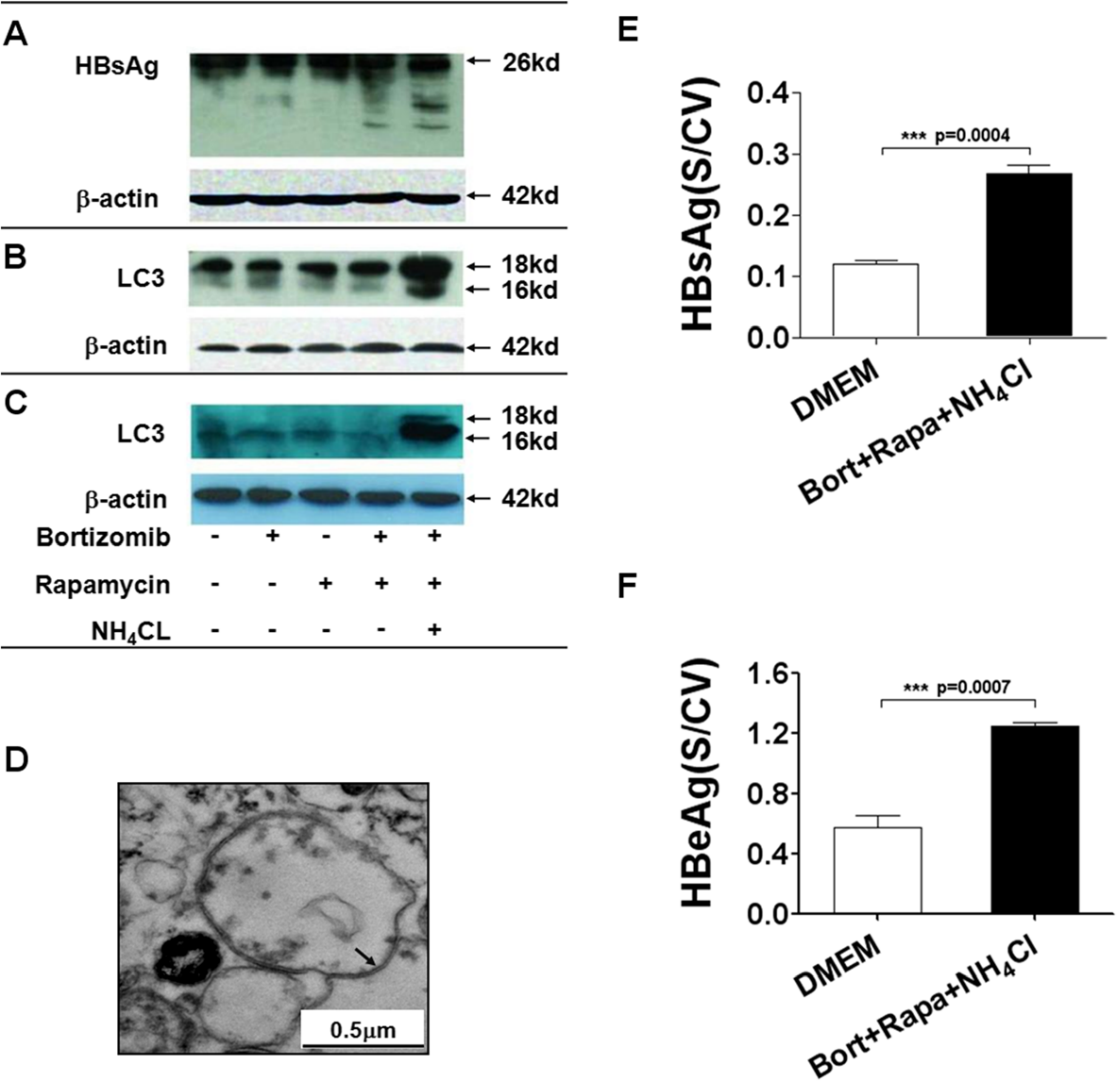
**Detection of HBV antigens in DRibbles.** HepG2.2.15 cells were treated without (lane 1) or with Bortizomib alone (lane 2), Rapamycin alone (lane 3), Bortizomib plus Rapamycin (lane 4) or combination of Bortizomib, Rapamycin and NH_4_Cl (lane 5) for 18 h. Lysates were prepared for the HBsAg and the LC3 conversion analysis by western blot **(A,B)**. The HBV^+^ DRibbles prepared from both untreated and treated cells were analyzed for LC-3-I and LC-3-II by western blot analysis **(C)**. Anti-β-actin antibody was included as a sample loading control. Morphology of DRibbles was observed by transmission electron microscopy **(D)**. The amount of HBeAg **(E)** and HBsAg **(F)** in the DRibbles were quantified by ELISA.

First, we investigated whether DRibbles contained HBV antigens when they were prepared from HepG2.2.15 cells that were transfected with HBV DNA and stably expressed HBV antigens. HepG2.2.15 cells were treated with Bortizomib, Rapamycin or NH_4_Cl at the pre-determined optimal concentrations; whole cell lysates and DRibbles were prepared as described and subjected to western blot analysis using antibodies against HBsAg and LC3 [13]. The major HBsAg protein did not differ after different treatments (Figure 1A). Interestingly, Bortizomib treatment led to appearance of HBsAg of lower molecular weight than full-length products and addition of NH_4_Cl further increased the appearance of these bands (Figure 1A). Because these bands appear only after inhibition of proteasome function, they are most likely representatives of DRiPs of HBsAg. No significant conversion of LC3 protein was found in HepG2.2.15 cells treated with Bortizomib, Rapamycin, or combination of both. However, accumulation of LC3-II in cell lysates and predominant LC3-II in the DRibbles was found when NH_4_Cl was included in addition to Bortizomib and Rapamycin treatment (Figure 1B,C) [20,21]. Transmission electron microscopy analysis showed that DRibbles had a double-membrane structure and their size was ranged from 100 to 1000 nm (Figure 1D). ELISA analysis showed that the amount of HBsAg and HBeAg in the DRibbles were doubled when all three drugs were combined (Figure 1E,F). These data indicated that combination of Bortizomib, Rapamycin, and NH_4_Cl could induce efficient sequestration of both HBsAg and HBeAg into autophagosomes.

### HBV^+^ DRibbles elicit protective HBV-specific immune responses

**Figure 2:**
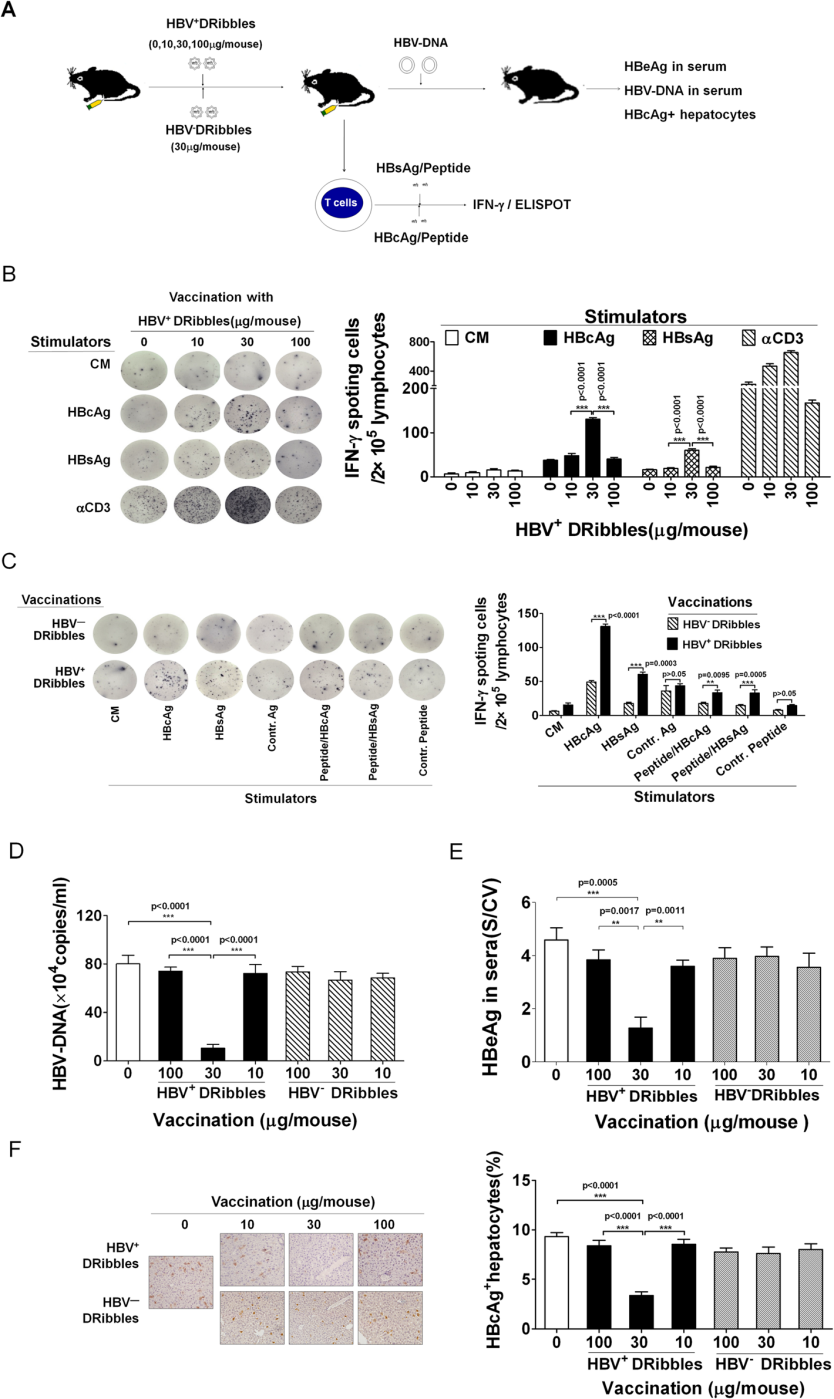
**HBV** ^**+**^ **DRibbles elicited HBV-specific protective immune response.** The schematic diagram outlines the experiment protocol **(A)**. The optimal dose of HBV^+^ DRibbles was determined by ELISPOT assay (n = 3). Lymphocytes from mice received HBV^+^ DRibbles generated a high number of IFN-γ secreting cells in 2 × 10^5^ lymphocytes **(B,C)**. Vaccinated mice were injected hydrodynamically with 10 μg of HBV plasmid DNA (n = 5) 7 days later. The serum HBV DNA **(D)** and HBeAg **(E)** was determined by real-time PCR and ELISA. Intrahepatic HBcAg was visualized by immunohistochemical staining (×200) **(F)**. Results represent three independent experiments.

To determine the optimal dose of HBV^+^ DRibbles to elicit HBV antigen-specific immune responses, different doses of HBV^+^ DRibbles or HBV^−^ DRibbles were administrated into the lymph nodes of naïve C57BL/6 mice (Figure 2A). We chose the intranodal injection of DRibbles as the route of antigen delivery because our preliminary experiments indicated direct injection of DRibbles into lymph nodes resulted the greatest DCs recruitment and T-cell activation. ELISPOT assay revealed that spleen and lymph nodes from mice injected with 30 μg of HBV^+^ DRibbles contained the highest number of HBsAg or HBcAg specific IFN-γ producing cells (Figure 2B). Injection of 10 μg of DRibbles was insufficient, while 100 μg showed a high-dose suppression. To further determine whether immunogenicity and xenoreaction of DRibbles derived from human HepG2 cells (HBV^−^ DRibbles) lead to immune reactive to HBV antigens, C57BL/6 mice were also immunized with titrated amount of HBV^−^ DRibbles. Noticeably, lymphocytes from 30 μg HBV^+^ DRibbles vaccinated mice generated much higher number of IFN-γ producing cells than that from 30 μg HBV^−^ DRibbles vaccinated mice when re-stimulated with either HBV antigens or HBV peptides (Figure 2C). Thus, HBV^+^ DRibbles effectively induced both HBsAg- and HBcAg-specific and HBc_129–140_, HBs_190–197_-specific cell-mediated immune responses. Thirty μg total proteins seem to be optimal dose for the HBV^+^ DRibbles vaccine administrated via intranodal injection in mice.

To evaluate whether 30 μg of HBV^+^ DRibbles could protect immunized mice from establishment of HBV replication via HBV plasmid DNA injection, C57BL/6 mice were vaccinated with PBS or different doses of HBV^+^ DRibbles or HBV^−^ DRibbles and were challenged with hydrodynamic injection of HBV DNA 7 days later (Figure 2A). Serum HBV DNA and HBeAg levels were used as the indicators of viral replication and production. Mice immunized with 30 μg HBV^+^ DRibbles, but not naïve mice or mice immunized with HBV^−^ DRibbles and other doses of HBV^+^ DRibbles, were protected from HBV DNA challenge (Figure 2D,E). To further corroborate this data, liver tissues were harvested and intrahepatic expressing of HBcAg was quantified by immunohistochemical staining. Similar to the results of HBeAg and HBV DNA, the percentage of HBcAg^+^ hepatocytes in the mice immunized with 30 μg HBV^+^ DRibbles was significantly lower than control mice injected with HBV^−^ DRibbles or PBS (Figure 2F). Moreover, there were no significant difference between the mice immunized with HBV^−^ DRibbles and those with PBS. Our results showed that HBV^+^ DRibbles vaccination, but not HBV^−^ DRibbles, could effectively reduce HBV replication and expression.

### CD8^+^ T cells induced by HBV^+^ DRibbles mediated suppression of HBV replication

**Figure 3:**
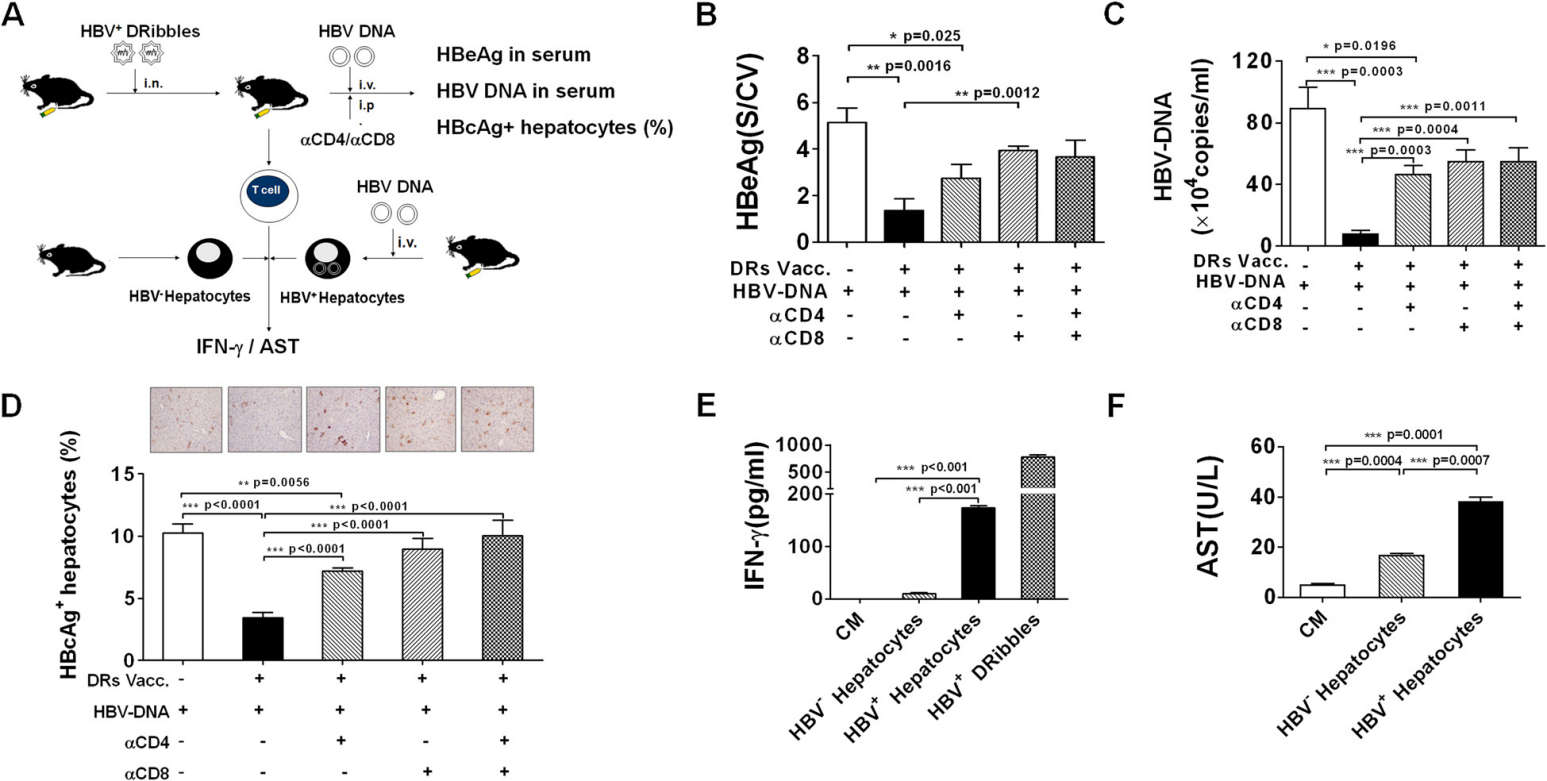
**CD8** ^**+**^ **T cells mediated protection induced by HBV** ^**+**^ **DRibbles.** The schematic diagram outlines the experiment protocol **(A)**. Mice were injected hydrodynamically with HBV plasmid DNA 7 days after vaccination and immediately subjected to intraperitoneal injection of PBS, anti-CD4 mAb, anti-CD8 mAb or anti-CD4 mAb plus anti-CD8 mAb, respectively (n = 5) **(B-D)**. The serum HBeAg **(B)** and HBV DNA **(C)** were detected by ELISA and real-time PCR. Intrahepatic HBcAg was visualized by immunohistochemistry (×200) **(D)**. Lymphocytes from HBV^+^ DRibbles vaccinated mice (n = 3) were co-incubated with target cells (the effector to target ratio was 100:1) before the supernatant IFN-γ **(E)** and AST **(F)** were assayed by ELISA and by clinical chemistry analyzer. These experiments were repeated three times with comparable results.

To investigate the requirement of CD4^+^ helper T cells or CD8^+^ effector T cells for the control of HBV replication, anti-CD4 mAb or/and anti-CD8 mAb were administrated intraperitoneally on the same day as HBV DNA injection into HBV^+^ DRibbles-vaccinated mice. The monoclonal antibodies were injected repeatedly every 3 days to maintain the depleted condition (Figure 3A). Results showed again that non-depleted mice immunized with HBV^+^ DRibbles had a reduced level of serum HBeAg (Figure 3B), DNA copy number (Figure 3C) and HBcAg^+^ expressing hepatocytes (Figure 3D). The DNA copy number in mice depleted of CD8^+^ effector T cells were not significantly different from that of control non-immunized naïve mice. Depletion of both CD4^+^ and CD8^+^ effector T cells did not further increase the serum HBeAg or DNA copy number beyond that of mice received anti-CD8 antibody (Figure 3B,C). These data indicated the critical requirement for priming of anti-HBV CD8^+^ T cells for viral clearance.

To examine the effect function of T cells from HBV^+^ DRibbles vaccinated mice, lymphocytes from HBV^+^ DRibbles immunized mice were co-cultured with hepatocytes isolated from HBV DNA injected mice (Figure 3A). Results showed that significantly higher levels of IFN-γ were detected in the culture supernatants when effector T cells were incubated with HBV-expressing hepatocytes than control hepatocytes (Figure 3E). Moreover, AST detection showed that significantly higher levels of AST enzyme release were found in HBV-expressing hepatocytes than control hepatocytes (Figure 3F). These data suggested that HBV^+^ DRibbles elicited strong cell immune response that was capable to produce IFN-γ and kill HBV-expressing hepatocytes isolated from mice *in vitro*.

### Vaccination with HBV^+^ DRibbles was effective against acute HBV infection

**Figure 4:**
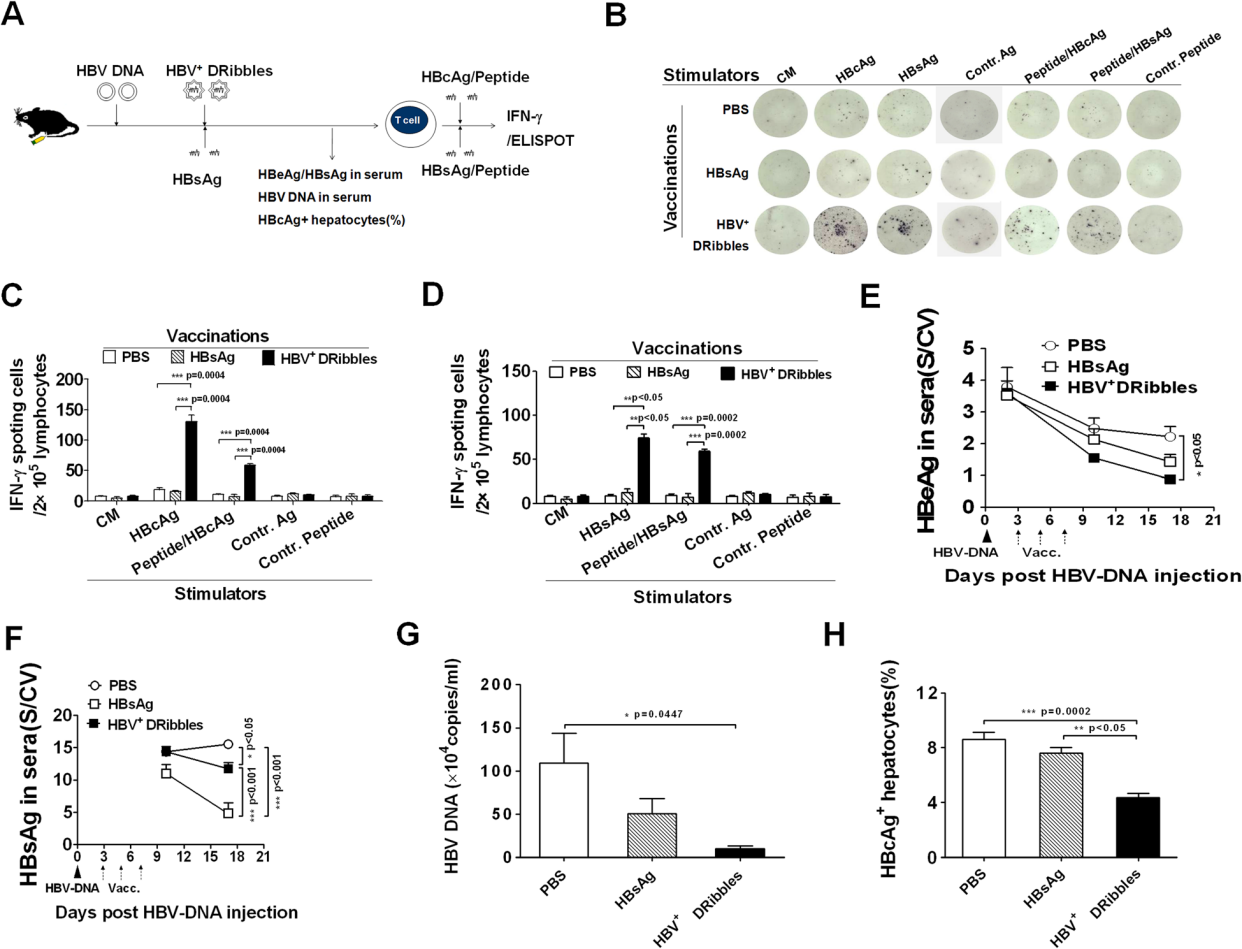
**Therapeutic efficacy of HBV** ^**+**^ **DRibbles vaccine.** The schematic diagram outlines the experiment protocol **(A)**. Lymphocytes from the acute HBV-infected mice received HBV^+^ DRibbles or HBsAg or PBS were isolated and re-stimulated with HBV antigens or peptides and the number of IFN-γ producing cells in 2 × 10^5^ lymphocytes was detected as described (n = 3) **(B-D)**. Serum HBeAg **(E)**, HBsAg **(F)**, HBV DNA copy number **(G)** and the percentage of HBcAg^+^ hepatocytes **(H)** of immunized mice or control mice were measured as mentioned above (n = 6). These experiments were repeated three times.

To test whether HBV^+^ DRibbles could be used as therapeutic vaccine in a setting that mimicking acute HBV infection and to compare the efficacy of HBV^+^ DRibbles with recombinant protein HBsAg vaccine, C57BL/6 mice were first injected with pAAV/HBV1.2 DNA to establish HBV acute infection and then were immunized with HBV^+^ DRibbles or HBsAg or PBS 2 days later. Two times boost immunizations were administrated by intramuscular injection every 2 days. Here, *α*-Al_2_O_3_ nanoparticles adjuvant was used in our HBV^+^ DRibbles vaccine due to its ability to greatly increase the efficiency of cross-presentation and anti-tumor response of cancer vaccines [19] (Figure 4A).

Consistent with previous results, lymphocytes harvested from HBV^+^ DRibbles-immunized mice generated a significantly higher number of IFN-γ producing cells than that from PBS or HBsAg immunized mice when re-stimulation *ex vivo* with HBV antigens or peptides (Figure 4B,C,D). The serum levels of HBeAg in HBV^+^ DRibbles-immunized mice were greatly reduced as compared to both HBsAg vaccinated mice and control mice, and no statistical difference was found between HBsAg vaccinated mice and the control mice (Figure 4E). We found that the levels of serum HBsAg in HBV^+^ DRibbles immunized mice were significantly lower than that of control mice without vaccination, but much higher than that of HBsAg immunized mice (Figure 4F). Detection of anti-HBsAg antibody in serum from immunized mice showed that HBsAg vaccination induced significantly higher levels of anti-HBs compared with HBV^+^ DRibbles vaccination (Additional file 1: Figure S1A). Serum anti-HBsAg antibody was detected only in two of six mice received HBV^+^ DRibbles immunization. Most importantly, HBV DNA copy number in the sera (Figure 4G) and percentage of HBcAg^+^ hepatocytes in the liver tissues (Figure 4H) in HBV^+^ DRibbles-immunized mice were greatly reduced as compared to control mice received either no vaccine or HBsAg vaccine. In addition, sera ALT and AST levels were determined and appeared not to be influenced by the vaccinations (Additional file 1: Figure S1B,C) and the livers from HBV^+^ DRibbles vaccinated mice showed normal architecture and a mild inflammatory responses (Additional file 1: Figure S1D). Thus far, our studies indicated that HBV^+^ DRibbles were more effectively than HBsAg, at eliciting therapeutic cell-mediated immune responses in an acute HBV infection mouse model established by hydrodynamic injection of HBV plasmid DNA and did not cause obvious liver damage.

### DRibbles induced therapeutic T cells and break tolerance in HBsAg tolerant mice

**Figure 5:**
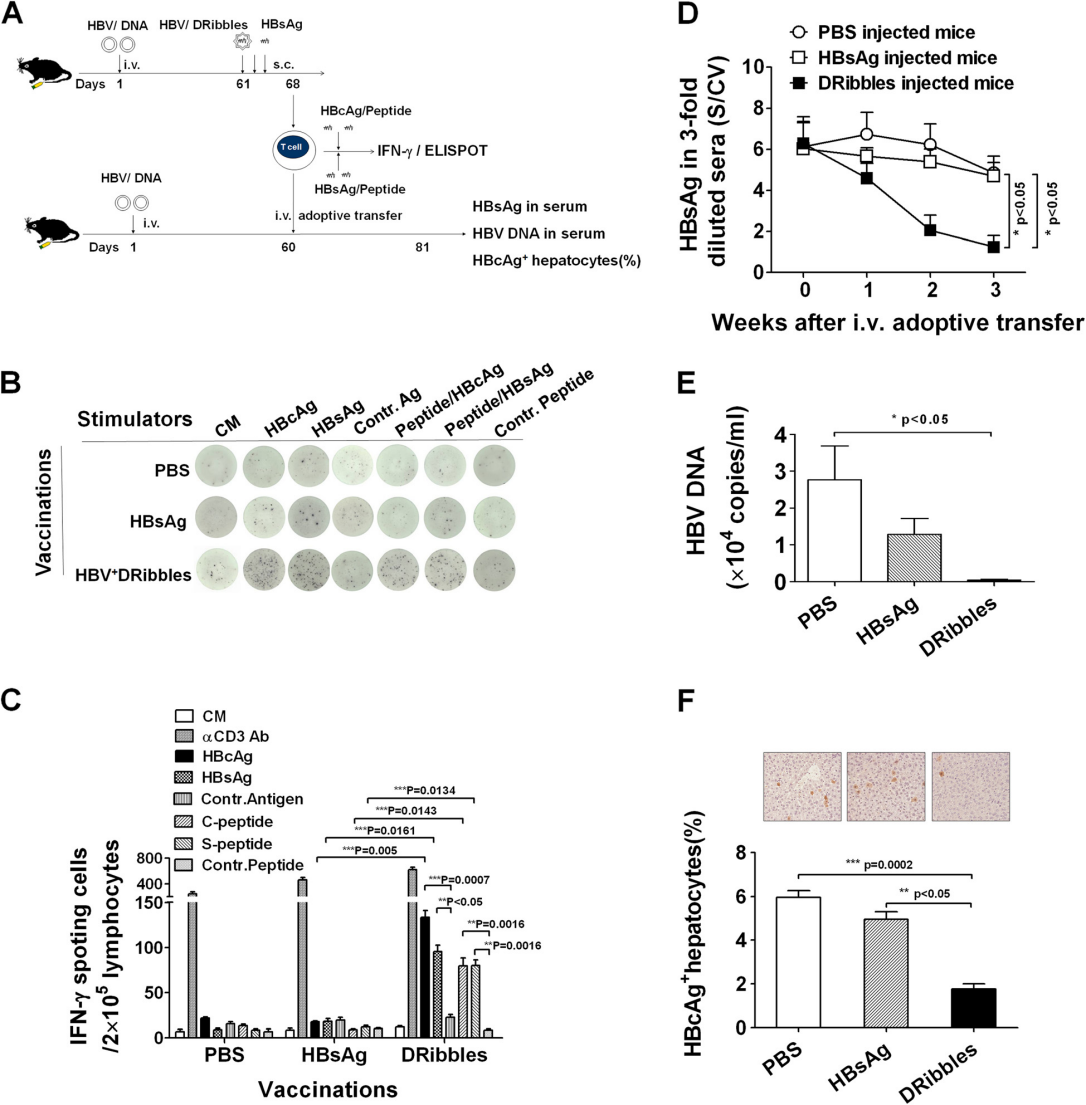
**Immunized lymphocytes transfer adoptive immunity against HBV.** The schematic diagram outlines the experiment protocol **(A)**. HBV^+^ DRibbles elicited IFN-γ producing cells in 2 × 10^5^ lymphocytes against multiple HBV antigens or peptides (n = 3) **(B,C)**. A total of 1 × 10^8^ lymphocytes were adoptively transferred intravenously into each of the new ‘HBV tolerant’ mouse. At day 7, 14 and 21 after adoptive transfer, serum samples were collected for detection of HBsAg **(D)**, HBV DNA copy number **(E)**. The liver tissues were harvested on day 21 and HBcAg^+^ hepatocytes were determined as described (n = 4) **(F)**. These experiments were repeated three times with comparable results.

Next, we investigated whether HBV^+^ DRibbles immunization could induce therapeutic immunity and break tolerance in a HBV tolerant mouse model. HBsAg tolerant mice were established by hydrodynamic injection of HBV DNA as described [18] (Additional file 2: Figure S2A). Two months after DNA injection, mice that are HBsAg positive were inoculated with HBV^+^ DRibbles or HBsAg or PBS. Seven days after first immunization, lymphocytes were harvested and re-stimulated by HBV antigens and peptides (Figure 5A). The data showed that HBV^+^ DRibbles, but not HBsAg, induced HBV-specific IFN-γ spot-forming cells when re-stimulated *ex vivo* with recombinant HBsAg or HBcAg proteins or MHC I binding peptides derived from these antigens (Figure 5B,C). Since the level of antibody response in DRibbles-vaccinated mice in the acute ‘HBV infected’ mouse model was very low, we decided to examine whether immune cells rather than serum conferred the control of HBV replication using an adoptive transfer model. To that end, immunized lymphocytes harvested from spleens and lymph nodes of immunized mice were adoptively transferred to a new set of HBsAg tolerant mice that received HBV DNA injection 60 days before. The levels of serum HBsAg, HBV DNA (Figure 5D,E) were markedly decreased and HBcAg^+^ hepatic cells were greatly reduced in HBsAg tolerant mice after adoptive transfer of lymphocytes from HBV^+^ DRibbles vaccinated mice (Figure 5F). In contrast, the mice received lymphocytes from HBsAg vaccinated mice did not exhibit any measurable effect. Interestingly, anti-HBsAg antibodies were detected in mice received lymphocytes from DRibbles, but not PBS and HBsAg immunized mice (Additional file 2: Figure S2B). Furthermore, the serum ALT and AST levels in the mice received lymphocytes from HBV^+^ DRibbles immunized mice were similar to that of received lymphocytes from non-immunized control mice (Additional file 3: Figure S3A,B) and the livers of the mice receiving HBV^+^ DRibbles-treated lymphocytes showed normal architecture with a mild inflammatory infiltrates (Additional file 3: Figure S3C). The results indicated that HBV^+^ DRibbles were able to reverse the tolerance to HBsAg and induce therapeutic T cells, and these HBV-specific T cells adoptively transferred into HBsAg tolerant mice did not cause severe liver damage.

## Discussion

Previously, others and we demonstrated that autophagy of antigen donor cells plays a critical role in the cross-presentation of tumor associated antigens or viral antigens [12-16]. In this study, we investigated whether multiple HBV antigens could be accumulated in DRibbles by treatment of HBV expressing cells with various agents modulating proteasome function and autophagy mediated protein degradation [13-16]. In addition to the accumulation of HBV antigens, DRibbles also contained specific ligands and ‘build-in adjuvant’ could stimulate specific subsets of DCs to efficiently cross-present antigens. It is likely that membrane-structured DRibbles might include innate adjuvant activity for activation of innate and induction of antigen-specific CD8^+^ T cell response [14].

The development of candidate therapeutic vaccines for patients with chronic HBV infection is greatly hampered by the lack of clinical relevant mouse models. Fortunately, when HBV DNA is artificially introduced into mouse hepatocytes, HBV expression and replication can be established. Different methodology has been exploited to introduce HBV genome into adult murine hepatocytes [18,22-24]. Hydrodynamic injection of plasmid DNA typically results in about 10% of HBV-expressing hepatocytes, long-term HBV expression and immunological tolerance similar to chronic human HBV carriers [18,23]. Hydrodynamic injection of HBV DNA of mice is proven to be a good murine model for studies of HBV replication and immune responses. Here, we employed this model to develop a novel HBV therapeutic vaccine based on autophagosomes derived from HBV expressing hepatoma cells.

The HBV^+^ DRibbles produced from a HBV expressing cell line were effective as a prophylactic vaccine or a therapeutic vaccine to treat mice with established HBV replication. Our results showed that HBV^+^ DRibbles could prime both HBsAg and HBcAg-specific immune responses and the IFN-γ producing and CD8^+^ T cells played dominant roles in the HBV clearance. It is previously reported that an impaired HBcAg-specific immunity is the major cause for HBV persistence after the hydrodynamic injection of HBV DNA [18]. HBcAg-specific immunity plays a critical role in the clearance of HBV and HBV antigen-harboring hepatocytes [25,26]. As expected, HBV^+^ DRibbles was able to reduce HBV replication *in vivo*. Whether HBV^+^ DRibbles could also induce immune responses to other HBV antigens, such as X protein and HBV polymerase remains to be determined. The HBxAg is of particularly relevant antigen as it is required for productive HBV infection and replication [27]. HBxAg is a short-lived protein and rapidly degraded by proteasomes [28] and X-protein specific T cells are less likely impaired or exhausted in patients with chronic HBV infection.

Previous studies have demonstrated that both CD4^+^ T cells and CD8^+^ T cells are required for elimination of HBV from the liver during natural infection [29,30]. As helper T cells for both B-cell and CTL responses against HBV, CD4^+^ T cells serve as master regulators of the adaptive immune response to HBV. B-cells produce critical neutralization antibodies that prevent viral entry and CD8^+^ T cells are the key cellular effectors mediating HBV clearance by killing of HBV-infected hepatocytes. We found that HBV clearance in our model primarily depended on CD8^+^ T cells and CD4^+^ T cells was less important. The predominant dependence on CD8^+^ T cells is probably because hydrodynamic injection of HBV DNA effectively bypassed the viral entry step. However, we did not examine whether HBV^+^ DRibbles could activate other innate immune components such as NK and NKT cells, as their contributions in the induction of specific anti-HBV immune responses and anti-HBV efficacy could not be ignored [31-33].

It is known that strong, multi-antigen specific, HBV-specific T cell responses in particular mediated by CD8^+^ cells are correlated with HBV control and resolution [29,30] and the neutralization antibodies could be critical important acting together with CTL to effective control and eliminate chronic HBV infection. During treatment of acute ‘HBV infection’ of our mouse model, HBV^+^ DRibbles vaccine was capable of eliciting strong and multi-specific T cell responses. Compared to HBV^+^ DRibbles vaccination, HBsAg immunization could not generate HBcAg- and HBsAg-specific and peptides-specific cell response; however, it was able to induce humoral response. Interestingly, some of the HBV^+^ DRibbles treated mice did produce anti-HBsAg antibodies, although the mean level was lower than that induced by HBsAg at the indicated time points. Importantly, when lymphocytes from immunized HBV tolerant mice adoptively transferred into new set of tolerant mice, the suppression of HBV DNA replication and the clearance of HBcAg^+^ hepatocytes were only detected in HBV^+^ DRibbles vaccinated group. Also, anti-HBsAg antibody response could be induced in some of the tolerant mice after adoptively transferred of lymphocytes from HBV^+^ DRibbles vaccinated mice. It is consistent with the notion that HBcAg-specific T cells support the anti-HBsAg antibody response [18]. The anti-HBsAg antibodies could also contribute to the neutralization of serum HBsAg, and the virus control and elimination of HBV infected cells required HBcAg-specific T cells.

*α*-Al_2_O_3_ nanoparticles, in contrast to traditional Alum adjuvant, are capable to deliver antigen for very efficient priming of CTLs and capable of boosting the anti-tumor efficacy of tumor-derived autophagosomes [19]. Our data demonstrated that HBV^+^ DRibbles mixed with *α*-Al_2_O_3_ nanoparticles could elicit an endogenous T-cell response capable of eliminating established ‘HBV infection’. The results suggested that HBV^+^ DRibbles vaccine together with potent adjuvant was capable to elicit therapeutic immune response and overcome HBV tolerance in our mouse model. The recent identification of HBV receptor makes it possible to generate authentic mouse model for chronic HBV infection [34].

Furthermore, DRibbles vaccine strategy was developed for cancer patients with terminal diseases [13-16], their safety and efficacy are tested currently in phase I and II clinical trials. A more stringent requirement for safety needed to be taken into consideration if DRibbles derived from a hepatocellular cell line will be tested in chronically HBV-infected humans. DRibbles isolated from cell line will unavoidably compose of cellular proteins in addition to HBV antigens. We did not observe cross-reactivity with normal cells when mice were vaccinated with DRibbles derived from cancer cells or cultured normal kidney cells and long term survival mice did not suffer noticeable autoimmune diseases in preclinical studies (unpublished data), but the potential long-term adverse effects of DRibbles vaccine in cancer patients are not known yet. Because of the high probability of developing HCC in patients with chronic HBV infection, the potential benefit of protection against HCC induced by HBV^+^ DRibbles derived from hepatocellular carcinoma cell line could outweigh the minimal risk of increased autoimmunity.

## Conclusions

Our results indicate that autophagosome-based vaccine could induce multifaceted immune responses and target T-cell mediated immune responses to many antigens of pathogens, which are not efficiently elicited or rendered tolerant during the natural course of infections. Thus, our findings have important implications for the development of therapeutic vaccines for the treatment of chronic infections of HBV or other viruses.

## Additional files

Additional file 1: Figure S1. Humoral response was induced in acute HBV infection. The same experiment protocol was performed as Figure 4. Serum samples were collected at day 14 after vaccination and anti-HBsAg antibody was detected by ELISA **(A)**. Serum ALT and AST levels were measured on automated clinical chemistry analyzer at indicated time points **(B,C)**. The liver sections were stained with hematoxylin-eosin at day 14 after vaccination (×400) (n = 6) **(D)**. Additional file 2: Figure S2. Tolerance toward HBsAg in HBV carrier mice. Serum anti-HBsAg antibody in HBV carrier (n = 4) or naive C57BL/6 (n = 4) mice after immunization with HBsAg vaccine (60 days after hydrodynamic injection of pAAV/HBV1.2 or PBS, the same protocol of HBsAg vaccination as above) was determined by ELISA at day 14 after vaccination **(A)**. Humoral response was detected in chronic HBV infection. The same experiment protocol was performed as Figure 5. Serum samples were collected at day 21 after adoptive transfer and anti-HBsAg antibody was assayed by ELISA (n = 4) **(B)**. Additional file 3: Figure S3. Serum ALT and AST levels were measured on automated clinical chemistry analyzer at indicated time points **(A,B)**. The liver sections were stained with hematoxylin-Eosin at day 21 after adoptive transfer (×400) (n = 4) **(C)**.

## Abbreviations

DRiPs: Defective ribosomal products
DRibbles: Autophagosome-based vaccine
HBV^+^ DRibbles: Autophagosome-based vaccine from HepG2.2.15 cells
HBV^−^ DRibbles: Autophagosome-based vaccine from HepG2 cells
