# Supplementary figures and images for: An autophagosome-based therapeutic vaccine for HBV infection: a preclinical evaluation
Source: J Transl Med. 2014 Dec 20;12:361. doi: 10.1186/s12967-014-0361-4 (PMC4301925; doi:10.1186/s12967-014-0361-4)

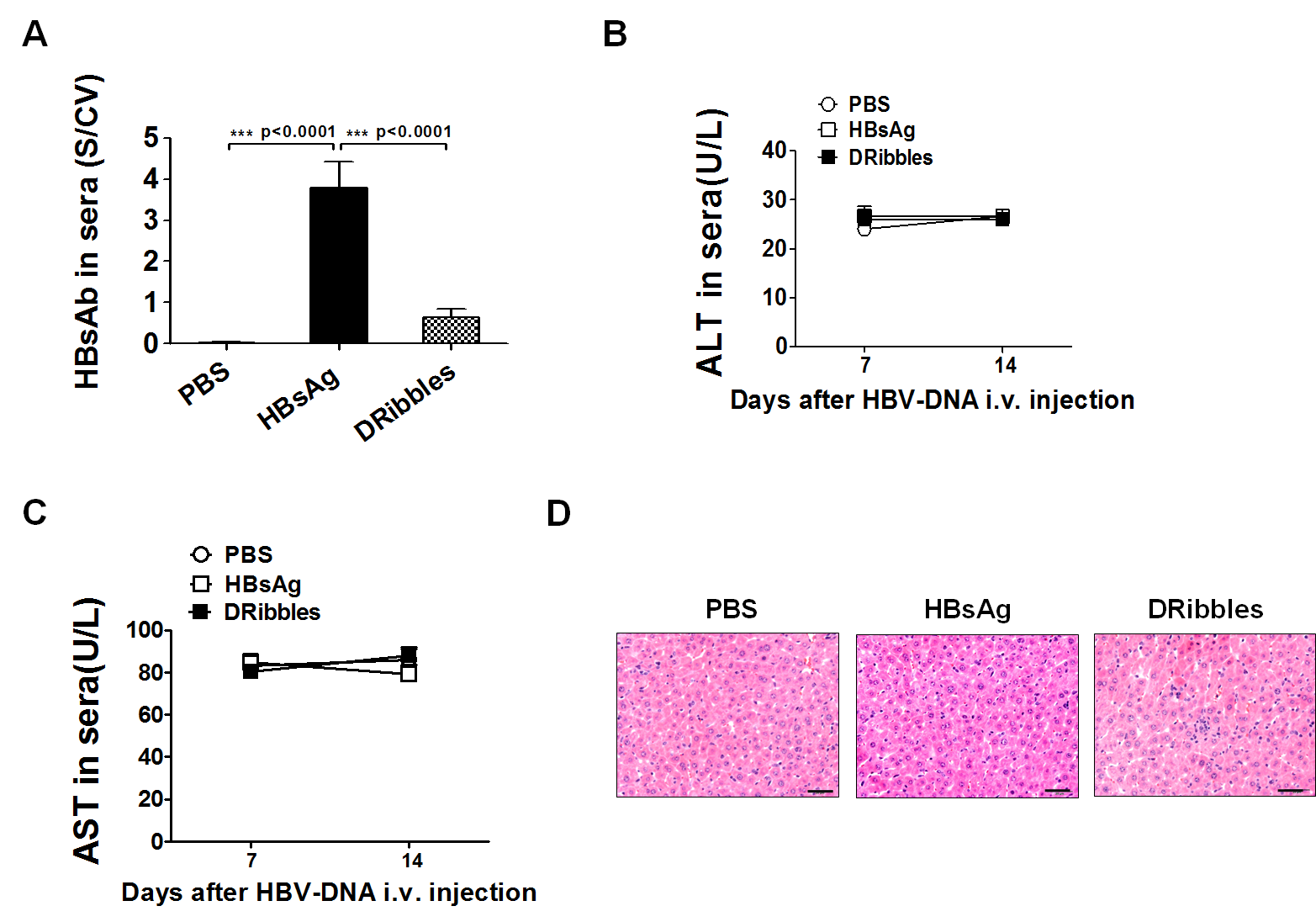

Supplement: Additional file 1: Figure S1. — Humoral response was induced in acute HBV infection. The same experiment protocol was performed as Figure 4. Serum samples were collected at day 14 after vaccination and anti-HBsAg antibody was detected by ELISA (A). Serum ALT and AST levels were measured on automated clinical chemistry analyzer at indicated time points (B,C). The liver sections were stained with hematoxylin-eosin at day 14 after vaccination (×400) (n = 6) (D). [file 12967_2014_361_MOESM1_ESM.tiff]

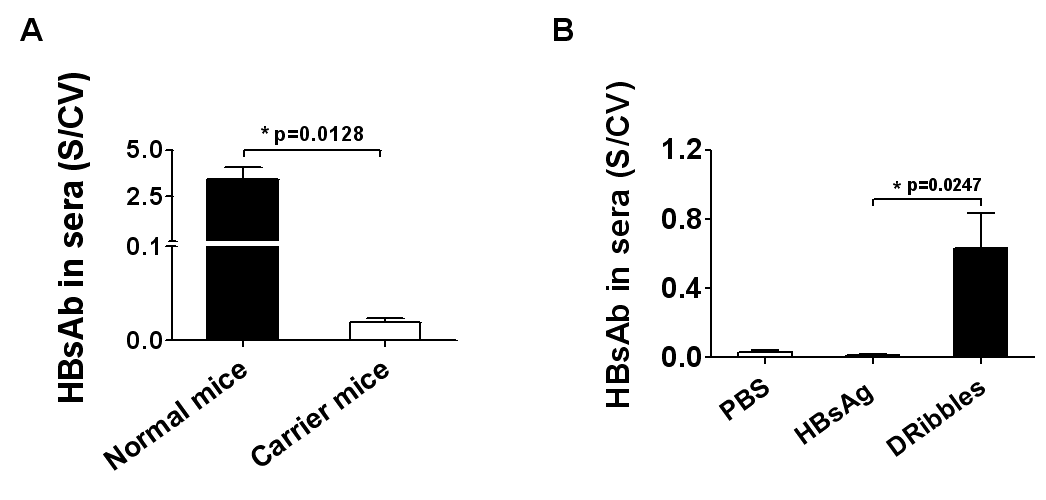

Supplement: Additional file 2: Figure S2. — Tolerance toward HBsAg in HBV carrier mice. Serum anti-HBsAg antibody in HBV carrier (n = 4) or naive C57BL/6 (n = 4) mice after immunization with HBsAg vaccine (60 days after hydrodynamic injection of pAAV/HBV1.2 or PBS, the same protocol of HBsAg vaccination as above) was determined by ELISA at day 14 after vaccination (A). Humoral response was detected in chronic HBV infection. The same experiment protocol was performed as Figure 5. Serum samples were collected at day 21 after adoptive transfer and anti-HBsAg antibody was assayed by ELISA (n = 4) (B). [file 12967_2014_361_MOESM2_ESM.tiff]

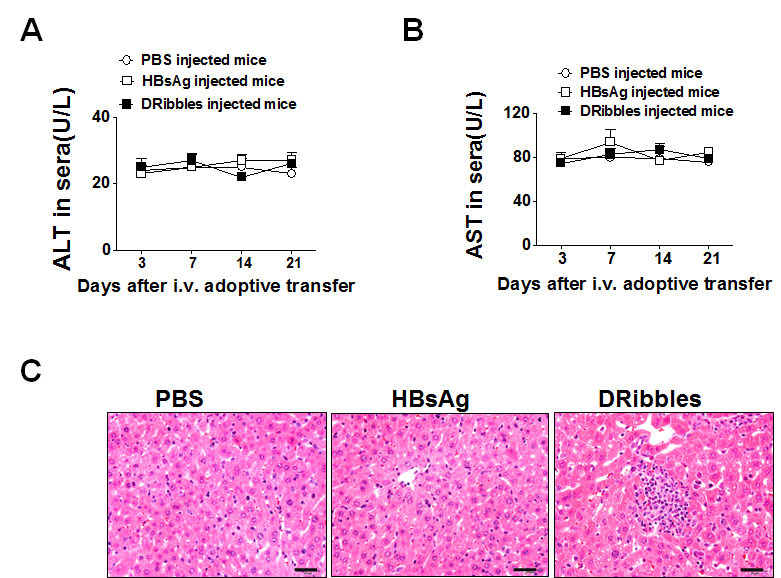

Supplement: Additional file 3: Figure S3. — Serum ALT and AST levels were measured on automated clinical chemistry analyzer at indicated time points (A,B). The liver sections were stained with hematoxylin-Eosin at day 21 after adoptive transfer (×400) (n = 4) (C). [file 12967_2014_361_MOESM3_ESM.tiff]
